# Intellectual property rights and the global exchange of molecular plant technologies

**DOI:** 10.1080/21645698.2026.2740254

**Published:** 2026-11-10

**Authors:** Aftab Haider, Asif Khan, András Szeberényi

**Affiliations:** a School of International Law, Southwest University of Political Science and Law, Chongqing, China; b Sarhad University of Science and IT, Peshawar, Pakistan; c International Arbitrator at Chongqing Arbitration Commission CQAC, China; d Global Research Fellow at ASTA Research Centre, Madagascar; e Sarhad Institute of Legal Studies, Sarhad University of Science and IT, Peshawar, Pakistan; f Department of International and Applied Economics, Széchenyi István University, Győr, Hungary

**Keywords:** Climate-resilient agriculture, Digital Sequence Information (dsi), intellectual property rights (ipr), molecular plant technologies, patent thickets

## Abstract

The global intellectual property regime for plants is structurally misaligned with the molecular tools on which climate-resilient agriculture now depends. As gene editing, synthetic biology, and Digital Sequence Information (DSI) become central to breeding drought-, heat-, and salinity-tolerant crops, these same technologies are increasingly enclosed within dense patent thickets, trade rules that go beyond the minimum standards of the Agreement on Trade-Related Aspects of Intellectual Property Rights (TRIPS), commonly called TRIPS-plus rules, and costly regulatory requirements. Using doctrinal legal analysis, a patent-landscape review, and product-level case studies, we show how this architecture generates a “Market Power Effect,” whereby a small group of firms can set prices, stack royalties, and control access to enabling technologies, effectively sidelining public breeders, small laboratories, and climate-vulnerable states. We develop the concept of “legal resilience” to capture the idea that molecular resilience in biotech crops is meaningless without legal structures that allow rapid, equitable diffusion of adaptive traits. Empirically, we link rising CRISPR-related patents, escalating licensing and compliance costs, and corporate concentration to stagnant yield growth, persistent human undernourishment, and an emerging form of digital biopiracy that existing access and benefit-sharing instruments cannot contain. We also examine the position of large middle-income innovators, China in particular, which do not fit the conventional North–South division and whose domestic policy choices are pushing toward stronger rather than weaker protection. On this basis, we propose a polycentric reform package: a climate-utility model tier for incremental local adaptations; a multilateral DSI benefit-sharing fund financed by small levies on commercial seed sales; compulsory humanitarian patent pools for abiotic stress-resistance technologies; regional molecular hubs that pair laboratories with pro-bono freedom-to-operate support; and sustained support for the existing network of genebanks and community seed systems as a practical counterweight to the hazard of genetic uniformity. Our central claim is that genetic resilience must be treated as an essential facility for human food security, not merely as a tradable private asset.

## 1. Introduction

The human-driven acceleration of climate instability has turned global agriculture into a front line of biological crisis.^1^ To withstand unprecedented abiotic stresses, the genetic resilience of staple crops must now be precisely reshaped using advanced molecular plant technologies.^2^ This evolving context contrasts with the stagnant world of international intellectual property law (hereinafter referred to as IP law). We are moving into an era in which crop improvement depends as much on genome editing and genomic data as it does on soil and seed.^3^ However, most of the legal regulations that we live under today were created in the twentieth century to protect mechanical inventions. They were not made to suit the dematerialized form of modern biology, where a plant’s value lies more in its digital information than in its physical form. The main argument of this paper is that our existing one-size-fits-all approach to patents is flawed. We suggest that the world needs a more polycentric structure, where genetic resilience is viewed not as a corporate secret, but as a human need.

Looking at the current legal environment, we see a system that is dominated by strong property-oriented logic. The rule is simple: when a company invests millions of dollars in molecular research, it has the right to 20 years of complete control to recover its investment.^4^ Although this approach has provided us with spectacular tools like Clustered Regularly Interspaced Short Palindromic Repeats (hereinafter referred to as CRISPR), it has also created patent thickets, a tangle of overlapping claims that makes it virtually impossible to work on local crop varieties in smaller research laboratories and then to commercialize them. Legally and logically, this creates what we hereby refer to as “Market Power Effect.” Large biotechnology companies prioritize highly profitable crops for wealthy markets and often overlook orphan crops that sustain millions of people in the Global South.^5^ The legislation treats a gene that confers drought tolerance like a software program or a car part, which ignores the fact that these genetic scripts are the backbone of agricultural production and hence human existence. We are literally constructing an IP law castle of Gothic magnificence and strength, but so amazing and so difficult to negotiate that even those who need the help cannot enter.

One of the major gaps in our present knowledge is the issue of the dematerialization of genetic resources. Previously, a researcher needed the actual seed of a plant to study it. Now all that one has to do is access its Digital Sequence Information (hereinafter referred to as DSI). A scientist is able to get a plant’s genetic blueprint, modify it in a laboratory, and develop a new trait without ever touching a physical plant from a foreign country. This prospect has left a massive gap in international treaties like the Nagoya Protocol, the 2010 supplementary agreement to the Convention on Biological Diversity that entered into force in 2014 and whose text is available from the CBD Secretariat.^6,7^ As a consequence, countries rich in biodiversity believe that their genetic heritage is being digitally exploited, while scientists fear that new laws will restrict life-saving research.^6^ In addition, there is a “Regulatory Wall.” It is not just about obtaining a patent license, but whether a developing country has the legal and technical absorptive capacity to actually utilize these molecular genetic tools. A national crop breeding program, for example, may be given the DNA sequence of a salt-tolerant rice, but the sequence on its own achieves nothing. Its crop scientists and breeders need a legal framework that lets them receive the material, transform and regenerate a line, backcross the trait into a locally adapted background, run multi-season field trials, clear the result through national biosafety review, and finally register and release the new crop line to farmers. Developing a GM or gene-edited line is a stepwise technical process rather than a single act of transfer, and each of those steps carries its own licensing and regulatory requirements. If any one of them is blocked, the technology stays on a shelf.

This study handles these issues by proposing a move to a step-by-step system of “Legal Resilience.” We argue that it is high time to stop believing that all genetic modifications should be patented with a utility term of 20 years. Rather, we ought to think of implementing the use of utility models, which are shorter and more adaptable protections that would give developing-country laboratories a reason to adapt globally available technologies to their own agricultural and environmental requirements. By drawing on both molecular biology and trade law, the study shows that protecting private innovation and feeding the world are goals that do not have to be in conflict. They are compatible, but only on the condition that we stop treating the genetic scripts on which our food system is based as property and start to view them as an essential facility to which everyone must have access in this dynamic time of increasing pressure upon agricultural systems.

To resolve these problems, this article has four main goals. First, it assesses why the existing utility patent system does not provide small-scale farmers with climate-ready characteristics. Second, it develops a viable way to disseminate the advantages of DSI in a way that honors the countries of provenance without restricting open science. Third, it discusses the justification of a “Climate Emergency Waiver,” a legal break-glass in case of emergency tool to be housed at the World Trade Organization (hereinafter referred to as WTO) that would enable countries to circumvent patents in times of food crisis. Lastly, it describes a proposal for “Regional Molecular Hubs.” These would be local collaborative hubs where scientists and lawyers work together to apply complex technologies under a shared freedom-to-operate framework, so that the future of food is not a series of corporate silos but rather a global network of collaborators.

## 2. Literature Review

In examining the development of legal systems to support crop innovation, Kjeldgaard and Marsh (1994)^8^ note that the diversification of intellectual property instruments – including utility and plant patents and trade-secret protections – has fundamentally transformed plant biology into a high-value economic resource. The need to select a proper IP protection system is no longer an administrative issue, but a strategic requirement. Scientific and molecular inventions are evolving at a higher rate than the legal systems that are meant to protect and enforce the respective rights involved. When discussing sovereignty and innovation, Correa^9^ states that even though international law explicitly acknowledges the sovereign rights of states over genetic resources, there is still confusion between tangible property and intangible assets. He argues that as developed countries extend patent rights over genetic sequences and microbial processes, the paradigm of genetic resources as a common heritage is being replaced by a proprietary paradigm, which disfavors nations that are rich in genetic resources but poor in the technology needed to use them.^9^

Helfer^10^ extended this functional view of IP in 2003, when she critiqued the International Treaty on Plant Genetic Resources to Food and Agriculture (ITPGRFA), referring to it as an effort to oppose privatization of the global genetic commons. The author emphasizes the contradiction between the seed wars of the 1980s and the modern global system based on a multilateral system, thus demonstrating that the delimitation between publicly available information and privatized genetic code is the central question in the international legal dispute. Also, the historical value of biodiversity hotspots has become more generally recognized. China, India, and Central Asia are important centres of origin for staple crops such as rice, soybean, and wheat, and have played a significant role in intellectual-property discussions concerning plant variety protection (PVP) and digital sequence information..^11^

Batur and Dedeurwaerdere (2014)^12^ explore the conflict between the communal character of agricultural heritage and the individualistic character of present-day patent law. They described an institutional mismatch. That is, patent law was not written for biology and long predates genetic engineering. Its basic architecture dates from the eighteenth century, and was built to give an inventor a limited monopoly in exchange for public disclosure, so that the time and money spent developing a machine or a process could be recovered. The difficulty is not that this bargain is wrong in itself. The difficulty is that it has been extended to living material, and largely through litigation rather than through legislation. In Diamond v. Chakrabarty (1980),^13^ the United States Supreme Court allowed a patent on a genetically engineered bacterium, and the grant of US Patent 4,736,866 to Harvard in 1988 for the OncoMouse made a transgenic mammal the first patented animal in any jurisdiction.^14,15^ Canada declined to follow this precedent, however, holding in Harvard College v. Canada (2002)^16^ that higher life forms are not inventions within the meaning of its Patent Act, which shows how unsettled the question remained even among closely related legal systems. Once the door to patenting of organisms was opened, however, the same reasoning was applied downwards to plant genes, promoters, and transformation methods. It is the rigid application of this framework to genetic engineering, and its subsequent reach back over mass selection and conventional breeding, that creates the problem that Batur and Dedeurwaerdere^12^ identified, because in those older practices the inventive contribution is cumulative and often collective rather than discrete. Patenting of GM crops produces partial stalling of the innovation process, especially in selective breeding programs that are based on on-farm monitoring and gradual genetic enhancement. The emphasis on strong property rights at the expense of collaborative agrobiodiversity utilization has effectively disenfranchised smallholder farmers, who have long been the custodians of plant resilience. The current legal paradigm presupposes a linear course of innovation, but molecular plant biology can be successful if based upon a circular, communal exchange of genetic material. The international framework, however, unintentionally creates a bottleneck that limits utilization of the agrobiodiversity needed to address complex abiotic stresses in a warming world because it does not provide a specific legal tool for collective innovation.^17^

The intensive development of molecular plant technologies has complicated the strict frames of the traditional patent law. Although patenting genetic sequences gives firms a reason to invest, it also allows single gene-encoded phenotypes to be enclosed within intellectual property protection, and it encourages the formation of patent thickets that crowd out smaller research organizations in developing countries.^18^ The main conflict lies in the need to balance the incentives given to molecular biologists with the necessity to consider life-saving crop adaptations as a global common good. Existing IP models do not deal well with individual climate-ready phenotypes, which are at once a humanitarian requirement and a valuable commercial asset. There is also a strong moral mandate for transfer of enabling technology in case of environmental crisis, which is to carefully handle the information of genetics, which is not yet well-defined in modern moral discourses. Lastly, the shift between the purely national protection of intellectual property and fair exchange of technology in the international arena will require the creation of a new legal norm, one that will recognize the issue of molecular stress tolerance as a common heritage of humanity.

The Nagoya Protocol has added a new layer of complexity to access and benefit-sharing (ABS) requirements, which directly affect the cross-border exchange of molecular data.^7,19^ Because ABS is the concept on which most of what follows in this study depends, it is worth describing it plainly. ABS is the principle that a country providing a genetic resource must be asked first, based on prior informed consent and mutually agreed terms, and is entitled to a fair and equitable share of whatever monetary or non-monetary benefit arises from the use of that genetic resource. Articles 5 and 6 of the Nagoya Protocol set out the terms for this bargain, and the wider literature traces how it has been implemented, and where it has failed, in practice.^7,19^ The transformation of physical biological matter into DSI forms a major gap in international law, since it allows persons to gain access to the genetic designs of heat-tolerant crops without triggering the customary ABS rules. The lack of connection between a physical sovereignty of a nation over its genetic resources and the borderlessness of the resulting molecular data is tangible. This legal gray area undermines trust between the countries that supply biodiversity and those that hold not only the molecular technologies for synthesis but also, and just as decisively, the technical capacity, the regulatory approvals, distribution networks, and capital required for commercial application. In the absence of an international agreement on DSI, the legal assurance needed to make large-scale investments in molecular plant technologies will never be achieved. The existing literature correctly pinpoints the issue, but falls short of suggesting an effective multilateral system that would automate the process of sharing benefits in the digital era.

When evaluating the institutional mechanisms of exploiting agricultural biodiversity, Smulders et al. (2021)^20^ argue that the IP systems should be redefined as not only the means of extracting profits, but also the regulatory systems that would guarantee the application of the Good Agricultural Practices. They also state that intellectual property rights can be used to implement sustainable agricultural practices like integrated pest management by making it a precondition to the issuance of a seed license.^20^ Dunwell (2005),^21^ in his overall review of plant transformation technology, lists the development of patent thickets as one of the main factors discouraging innovation in the agricultural sector. The paper shows that, although many individual patents may have little commercial potential, a small set of key patents on selectable markers and promoters can effectively preclude a newcomer’s freedom to operate.^21^ The agglomeration of proprietary rights has long been the trigger for the concentration of the agricultural biotechnology industry, forcing small firms into large, and even unfair, cross-licensing deals. The author argues that this legal overload hinders the decentralized process of developing climate-resilient crops, because the transaction costs incurred in moving across intersecting intellectual property rights are greater than the potential research gains that public institutions could offer.^21^ The structural weakness identified by the study is critical. The legal system rewards the first party to lock up a molecular tool, despite the lock being an obstacle to further uses of the tool in the realization of global food security goals. Without a more flexible open-source attitude to the basic molecular methodologies, the current legal environment will remain biased toward industrial-scale production at the expense of scientific responsiveness to the needs of smallholder farmers.

Kowalski et al. (2002)^22^ provide a methodological basis for the deconstruction of transgenic plants into their IP parts, which are selectable markers, promoters, structural genes, etc. Even though the technology has been developed since the early days of transgenics to CRISPR, their framework cannot be ignored when it comes to the identification of Freedom to Operate (FTO). Freedom to operate is the conclusion, normally reached after a formal legal search, that a product can be made, used, or sold in a particular territory without infringing anyone else’s valid intellectual property rights. It is a different question from whether the product is itself patentable, and it is the question that in practice decides whether a variety reaches practical utilization by a farmer. We have applied this method of analysis to modern CRISPR-edited crops, where a single abiotic stress-resistant crop could have over twenty different intellectual property elements, running from the promoter and the selectable marker through to guide RNAs, the editing enzyme, and the modified trait sequence itself. Wyse and Luria (2021)^23^ examine what they termed the upstream-midstream-downstream paradigm of innovation. In that framework, upstream refers to the enabling discoveries and genetic traits, midstream to the tools and processes that convert a trait into a working line, and downstream to formulation, delivery, and the finished commercial product itself. Each stage can be patented separately, and each therefore adds a further layer to the license stack. This model is especially relevant to the study of abiotic stress resistant crops, with high-value specialized crops (such as medical cannabis or climate-resistant pulses) experiencing an influx of patents in both the Downstream category (delivery and formulation), and the Upstream category (genetic traits).^23^ Applications grew by 0.6% in 2024 to reach 29,250 (Figure 1).

**Figure 1.**
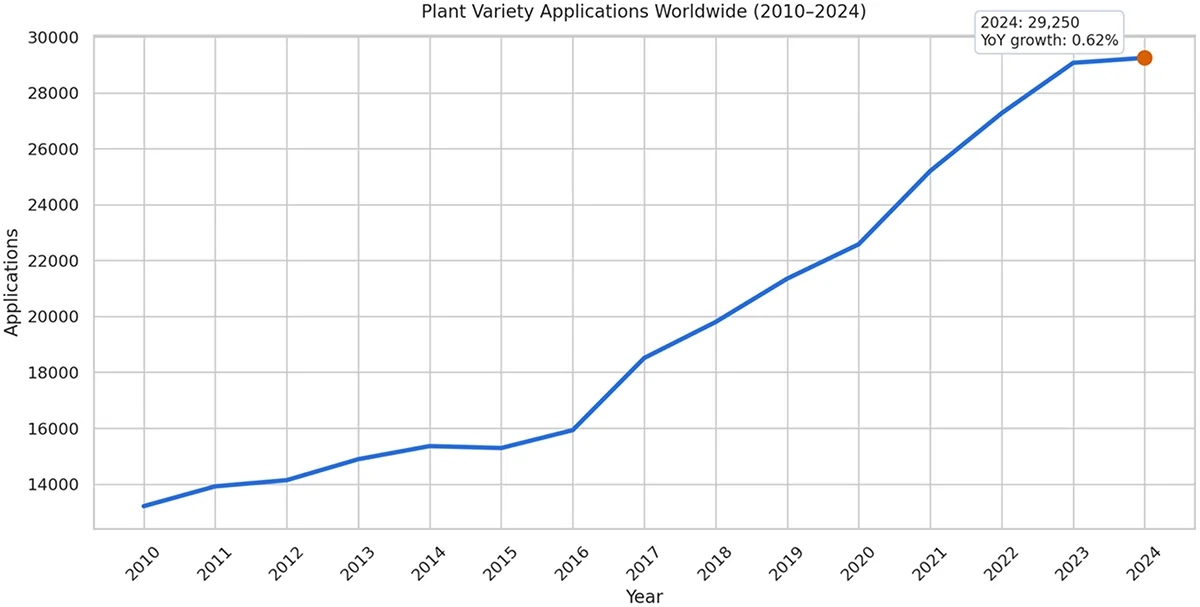
Plant variety applications worldwide, 2010–2024.

Applications grew by 0.6% in 2024 to reach 29,250. Source^24^:

The global CRISPR in agriculture market valuation is estimated at USD 1.12 billion in 2024, and USD 1.34 billion by the end of 2025. The sector is projected to grow, reaching USD 3.26 billion by 2030 (Figure 2), with a compound annual growth rate (CAGR), that is the smoothed year-on-year rate at which a value would have to grow to move from its starting to its ending figure over a given period, of 19.4% between 2025 and 2030. Source^25^:

**Figure 2.**
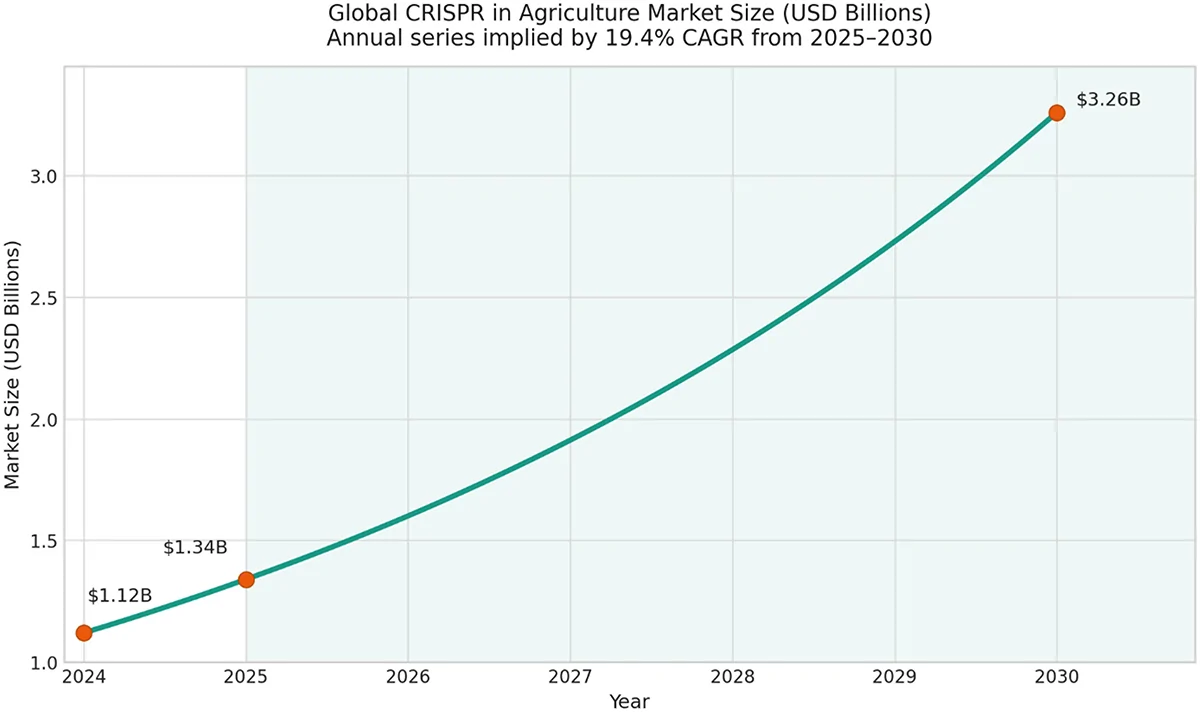
Global CRISPR in agriculture market value (USD billions).

The Climate-Resilient Crop Breeding Market size was estimated at USD 2.93 billion in 2024 and expected to reach USD 3.16 billion in 2025, at a CAGR of 8.38%, to reach USD 5.59 billion by 2032 (Figure 3). Source:^26^

**Figure 3.**
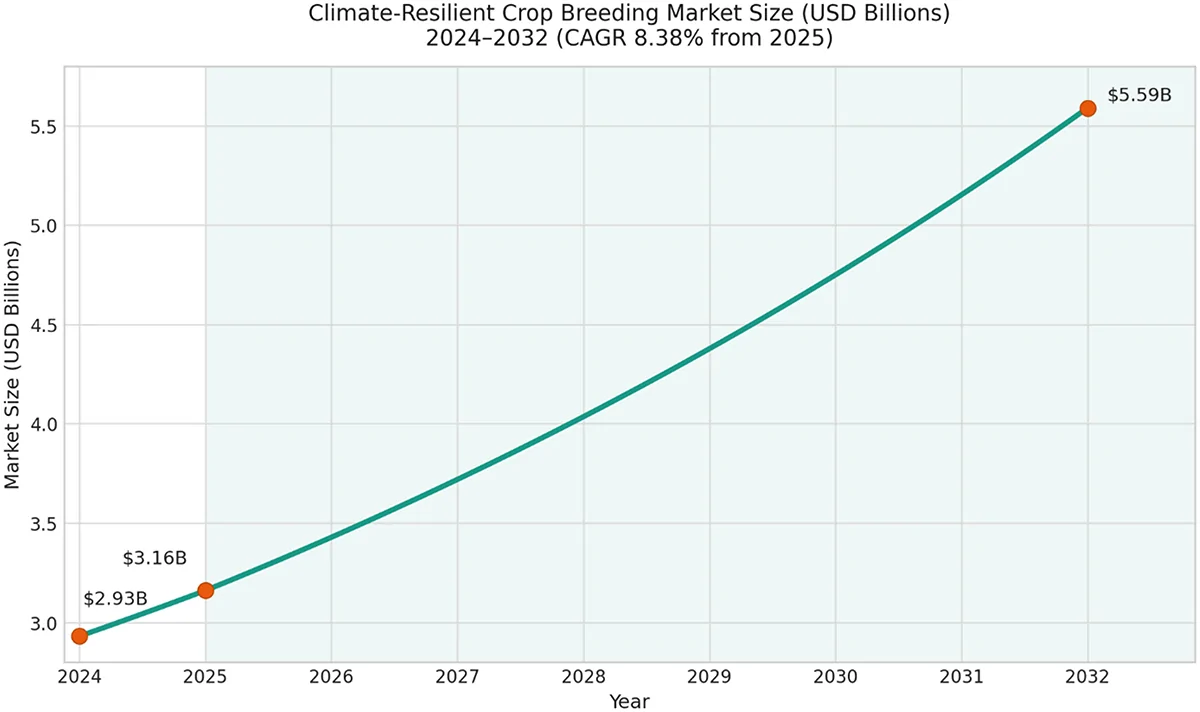
Climate-resilient crop breeding market value (USD billions).

In Figure 4(a), FAO reports that nearly 1.4 billion hectares of land (just over 10% of the total global land area) are already impacted by salinity, with an additional one billion hectares at risk due to the climate crisis and human mismanagement.^27^

**Figure 4.**
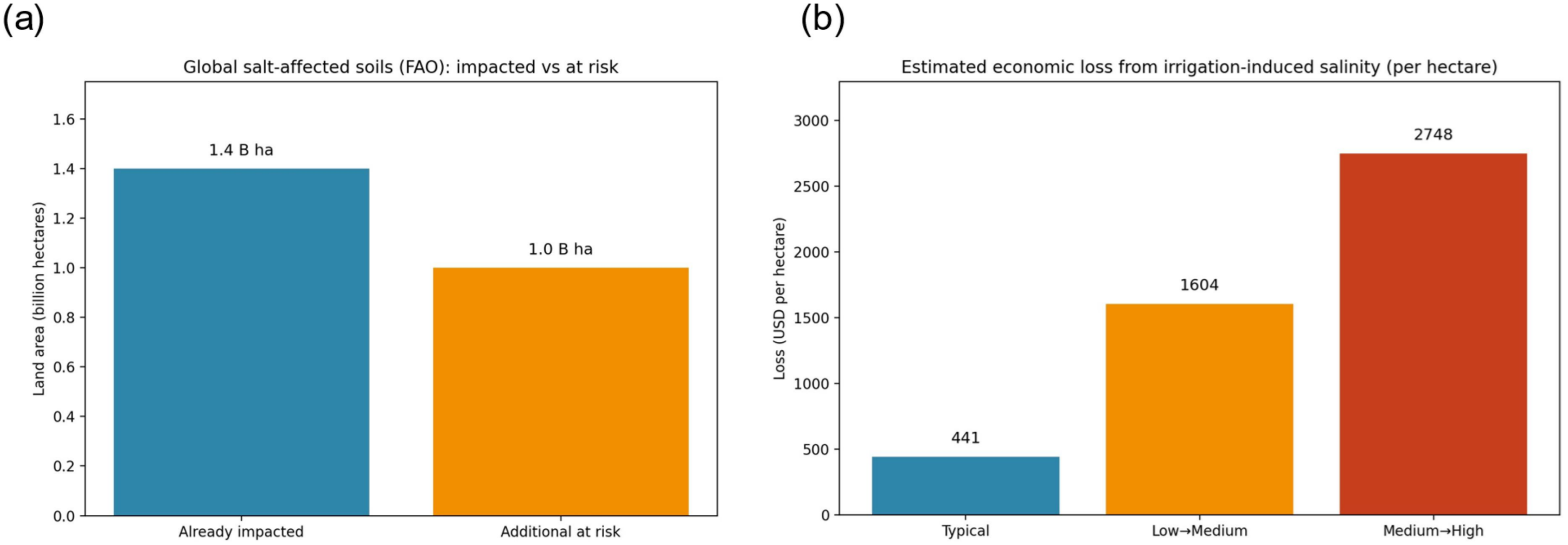
Impacts of salinization of soil (a) global salt-affected soil: impacted vs at risk (b) estimated economic loss from irrigation-induced salinity (per hectare).

In Figure 4(b), the extrapolation of global loss due to irrigation-induced salinity is roughly estimated as USD ~27.3 billion per year, caused by reduced crop production (approximating USD 441 loss per hectare of land). Recent studies indicate that the economic loss could be as high as USD 1604 and USD 2748 per hectare if the salinity levels rise from low to medium and medium to high, respectively, and the gross profit in undertaking any migratory measures might be too low for the farmers to adopt sustainable irrigation strategies. Comparable to the above inference, a study in Australia estimated that an expenditure of AUD 65 billion over 10 years would be required to reclaim the land degradation caused by salinity and sodicity in the country.^28^

When considering the legal and economic structure of contemporary agriculture, Rapela (2020)^29^ argues that a major paradox has been created. On his account, the shift toward “Open Innovation” has not delivered the increase in crop productivity that was expected of it. That claim needs qualification, and we report it rather than endorse it. Global yields of the major staples have continued to rise, and in the industrialized systems where GM varieties are widely grown they have risen appreciably. Rapela’s argument is better read as being about the rate and the distribution of those gains rather than about their existence, since yield growth has been concentrated in a small number of commodity crops and high-margin markets and remains well below the rate that would be needed to meet projected demand. He claims that the existing IP system promotes a strategic reduction of the genetic base, with breeders depending more on a limited number of elite, guarded lines to guarantee legal adherence and market leadership, at the cost of maintaining wider genetic variation.^29^ In addition to this analysis, Galushko (2012)^30^ gives an empirical analysis of how these property rights influence international trade, especially the contradiction between the market-expansion effect and the market-power effect. Her work reveals that despite the need to have strong legal protection to enable the transfer of technologies that are easily duplicated, like genetically modified crops, it is more likely to create a market-power effect. In such situations, exporters can limit the supply of seeds to ensure monopoly rents.^30^ The combination of these views brings out an imbalance in the structure of international law, in that the high-standard protection desired by the TRIPS-plus provisions may inadvertently cause technological bottlenecks that stifle the very food-security goals that they are expected to enhance (see Table 1 for a comparative summary of the impact of stronger IPR across different crop categories).

**Table 1.** Impact of stronger intellectual property rights on different crop categories.

Crop Category	Impact of Stronger IPR	Reasoning
Genetically Modified (GM)	High Impact	TRIPS provisions are crucial for facilitating the transfer of GM technology, as it is easily imitated.
Hybrid Crops	Low/Negative Impact	IPR is less critical because hybrids have built-in “biological protection,” as farmers cannot save and replant seeds with the same vigor.
Open-Pollinated Crops	Variable	Stronger IPR often results in a “Market Power” effect, where exports may actually decrease due to high pricing and restricted access.

Note: That Table 1 is our own synthesis rather than new empirical data. The crop categories and the direction of effect are derived from Galushko (2012),^30^ who distinguishes the market-expansion effect from the market-power effect in U.S. seed exports, and from Zhou, Sheldon and Eum (2018),^31^ who examine how far TRIPS accession translates into actual technology transfer. The reasoning column summarizes the mechanism that each of those studies proposes; the underlying difference between the respective categories is the reproductive biology of the plant, since hybrids carry a degree of built-in biological appropriability that open-pollinated and transgenic material does not. Zhou and Sheldon (2018)^31^ show that the adoption of WTO-TRIPS standards has a positive influence on the level of U.S. field crop seed exports. They argue that while participation in international IP agreements facilitates the transfer of agricultural technology, the actual effectiveness of this transfer is often limited by accompanying policies on foreign direct investment and local licensing, which firms use as alternatives to direct trade.^31^

Louwaars (2006)^32^ approaches the same problem institutionally. By this we mean that he asks how the rules, organizations, and enforcement practices surrounding a technology determine who can use it, rather than asking only what the technology is capable of doing. He criticizes the one-size-fits-all approach to international IP, indicating that the existing legal paradigm does not facilitate equity between countries or enhance the livelihoods of people in developing countries. He provides an outlook of joint IPR strategies and humanitarian license systems, including the ones that were first developed by the Generation Challenge Programme^33^ and the Public Intellectual Property Resource for Agriculture,^34^ to make sure that the sharing of genetic resources is not closed to the public sector.^32^ The seeds market has grown strongly in recent years, and is forecast to grow from $74.13 billion in 2025 to $78.44 billion in 2026, a compound annual growth rate (CAGR) of 5.8%.^35^ Most of this value is captured by countries with developed TRIPS-plus applications, leaving many of the climate-sensitive areas of the Global South with limited access to the most recent molecular adaptations.^36^ Also, according to recent World Intellectual Property Organization (WIPO) statistics, despite the 14% increase in open-source patent applications, the cost of cross-licensing of key transgenic traits has increased by 12% per year, which even the strongest trade agreements cannot overcome.^37^ We believe that the results lead to a paradigm shift in the theory of international law, where appropriability, meaning the extent to which an innovator can capture the commercial value of an invention rather than see it flow to imitators, has to be balanced with the need to have freedom to innovate that safeguards the collaborative character of development of agrobiodiversity.

Still on this theme of legal diffusion around the globe, Welsh et al. (2021)^38^ discovered that the containment tendencies of IP that originated in the United States have spread to both OECD and non-OECD nations, creating a uniform, but frequently rigid worldwide research setting in plant science. Their study shows that despite the intent that international economic agreements harmonize protections, they often overlap with national policies in a way that has the potential to limit the freedoms of local scientists and academic institutions.^38^ The World Intellectual Property Organization (WIPO)^24^ reports that plant variety applications worldwide have continued to rise, reaching 29,250 filings in 2024. Over the same period, the yield penalty associated with global warming has been quantified with increasing confidence. In the absence of climate adaptation, CO_2_ rise, and genetic improvement, each additional degree Celsius of global mean warming is estimated to reduce global yields of wheat by 6.0%, rice by 3.2%, maize by 7.4%, and soybean by 3.1%.^39,40^ We are not claiming that the growth in filings causes the yield penalty. Our point is that a rising count of protected varieties is a poor proxy for progress on crop adaptation, because it records legal activity rather than agronomic outcome, and it says nothing about whether the traits farmers need are reaching the fields where global warming is being felt hardest. Read that way, the two trends suggest that legal effort and biological need are not tracking one another, and it is that divergence, rather than any causal relationship between the numbers, with which this study is concerned. This implies that IP legal experts, influenced by the ideology of academic capitalism, may lay more emphasis on the number of patents than on the urgent need to spread technologies transnationally.

Ferrari et al.^41^ assert that IPRs are the main institutional tools of enforcement of the appropriability of innovations embodied in transgenic seeds. Their analysis of the industry recognizes that high-stakes infringement litigation is a strategic tool used by dominant firms to create barriers to entry and technological spillovers that would otherwise favor farmers or other competitors.^41^ The most recent publication by Cognitive Market Research shows that the global market for genetically modified seeds is estimated to have reached $30.1 billion in 2022 and is projected to reach $48.79 billion by 2030. The sector is projected to grow at 9.8% per year over 2023–2030, while corporate legal costs for patent protection are rising by an average of 8.5% per year.^42^ Estimates suggest that investments worth $3.78 billion to date have been made in CRISPR-utilizing startups. As CRISPR-Cas continues to revolutionize the life sciences, it also presents a complex and ever-expanding IP landscape, with more than 11,000 patent families already filed.^43^ A patent family is the set of applications and grants filed in different countries that all claim the same underlying invention, so the 11,000 figure reflects distinct inventions rather than distinct national documents. On the question of who holds the rights, the position is genuinely unsettled rather than resolved, and it differs by jurisdiction. Doudna and Charpentier received the 2020 Nobel Prize in Chemistry for the discovery, and their institutions, referred to collectively as CVC, hold the foundational European patents. In the United States, however, the outcome has so far run the other way. The Broad Institute holds the contested claims to the use of CRISPR-Cas9 in eukaryotic cells; the Court of Appeals for the Federal Circuit remanded the priority question in May 2025 on the ground that the Patent Trial and Appeal Board had applied the wrong standard for conception,^44^ and on reconsideration the Board reaffirmed Broad’s priority in March 2026.^45^ Parallel disputes involving other parties remain pending. Scientific credit and legal title therefore do not sit in the same place, and that gap is itself part of the problem that this study describes: a breeder in a developing country seeking a license must currently negotiate with different rights holders depending on where the crop will be grown and where it will be sold. We believe that these results point to a systemic problem that is becoming critical. The legal cost of innovation is beginning to erode the biological usefulness of the solutions being produced, which slows the deployment of the molecular tools that are needed at a time when climatic volatility is increasing. Jarvie (2015)^46^ argues that today’s rules for protecting plant varieties – both traditional and genetically modified – do not clearly support two key goals: increasing crop yields and protecting biodiversity. He suggests the implementation of the so-called utility models as a pragmatic alternative to the inflexible frameworks of the International Union for the Protection of New Varieties of Plants (UPOV) and the Agreement on Trade-Related Aspects of Intellectual Property Rights (TRIPS). Such a change would help avoid the necessity of grand international reforms and would be more appropriate to facilitate the commercial breeding research that would be required to achieve long-term stability in the food supply.^46^

This necessity of balanced IP protection is also reflected in the works of Le Buanec and Ricroch (2021)^47^ who state that although the high costs and risks of plant biotechnology make the protection of breeders a necessity, the instruments should be well-designed to provide future generations with a greater variety of genetic material. They point out that in Europe, the protection afforded by patents to biotechnological inventions is extended to the transgenic varieties into which they are transferred, but they insist that farm-saved seed and breeder privilege are essential to the continued ability of public and private research to drive innovation.^47^ Regulatory compliance for a single new transgenic variety currently averages around USD 136 million, a financial barrier that in effect confines the development of specialized transgenic varieties, particularly for minor and orphan crops, to a handful of multinational companies.^48^ We believe that these points highlight a growing tension between the commercial interest of appropriability and the humanitarian interest of making technology accessible, which suggests that the IP landscape is still more concerned with the safety of existing investment than with the pressing biological adaptations needed to secure the advancement of world agriculture.

This issue is informed by economic context, as explained by Giannakas (2003),^49^ who provides a discussion on the issue of IP infringement in developing nations under the TRIPS Agreement. He argues that the illegal use of safeguarded biotechnologies in such areas is often a logical economic reaction to excessive monopoly prices and ineffective enforcement systems, and that the welfare consequences of IP are very contingent on whether a nation is a net innovator or a net technology consumer.^49^ A note on dating is necessary at this point. The 2026 agricultural year is still in progress at the time of writing, so no full-year 2026 figures exist. Everything reported here for 2026 is either a value reported up to the first quarter of that year or a published forecast, and it is identified as such throughout. On that basis, figures published in early 2026 for the preceding season put losses from soybean seed piracy in Brazil alone at USD 1.76 billion per year, against enforcement spending by the largest biotechnology firms running into the hundreds of millions.^50,51^ Reporting through the first quarter of 2026 indicates that the global seed and biotechnology market continues to grow, with the fastest growth in climate-adaptive traits in staple grains. These indicators are summarized in Figure 5, which reports the position as of March 2026. Utility patents on gene-edited plants have increased 19% annually in the United States, as a shift to CRISPR-based alterations is associated with much higher licensing costs than conventional transgenic traits.^55^ Commercial seed market reporting published in early 2026 puts the average price increase for high-yield hybrid maize in emerging markets at 9.4% year-on-year, which is attributed in part to the growing implementation of TRIPS-plus obligations in bilateral free trade agreements.^56^ In the meantime, WIPO reports that the number of plant variety protection applications in Asia has surpassed that in Europe for the second consecutive year, marking a shift in the geographic focus of crop innovation.^57^

**Figure 5.**
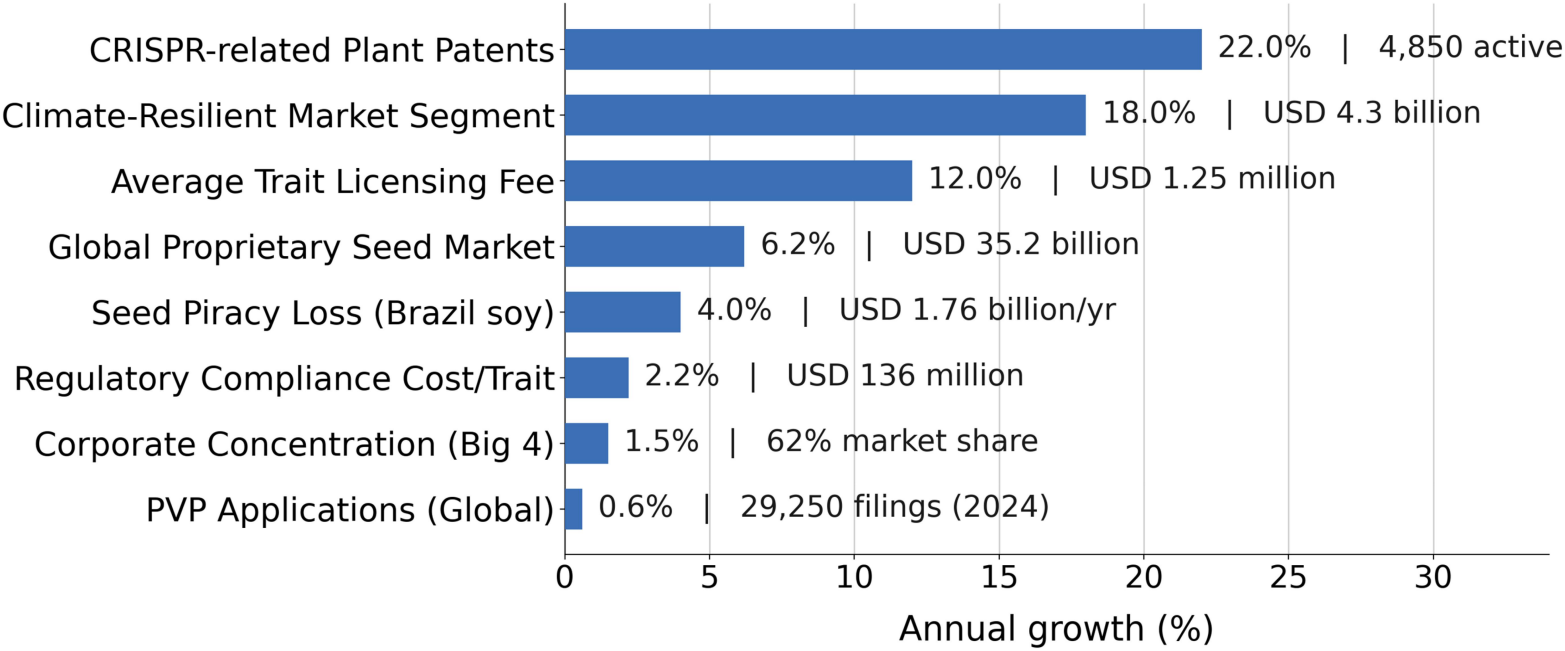
Key indicators of the global proprietary seed and biotechnology market (data as of March 2026).

Sources^51–54^.

That said, these new measures indicate a legal decoupling in which the expansion of IPR tools is not triggering the requisite biological breakthroughs in climate resilience. The emergence of a new form of biopiracy, digital biopiracy, where genetic sequences in locations of diversity are patented without physical possession, has resulted in a rise in international benefit-sharing conflicts under the Nagoya Protocol.^58^ Since cross-licensing costs for basic breeding technologies are now consuming a significant share of overall research spending by mid-sized companies, the blocking power of established patent holders continues to centralize the industry.^59^ Data reported through early 2026 indicate that the four largest agribusiness groups now account for around 62% of the world’s proprietary seed market.^60^ As a result, unless there is a shift to humanitarian licensing models and utility models championed by modern jurists, the legal structure of agriculture will be a hindrance to the very transboundary cooperation that is needed to adapt to ongoing environmental turbulence.

## 3. Methodology

To solve issues posed by the complex nexus of molecular plant science, IP law, and global food security, this study takes a hybrid approach, which integrates: (a) doctrinal legal analysis, (b) empirical review of the patent landscape, and (c) case study research. The general aim is not to build a single model of any one kind, whether econometric, legal, or institutional, but to assemble a logically consistent, multi-level evidentiary corpus that draws on all three. The econometric material indicates what the market is doing, the doctrinal material indicates what the law permits, and the institutional material indicates whether a given country is in a position to act on either. Taken together, they can support a normative proposal for a polycentric, resilience-oriented IP framework. The research design is implemented in four overlapping layers: (a) a patent landscape and concentration mapping exercise on climate-resilient characteristics, (b) a comparative legal analysis of TRIPS-plus standards in regional and bilateral trade agreements, (c) a qualitative synthesis of Freedom to Operate (FTO) and DSI governance barriers facing public-sector and Global South breeders, and (d) a sequence of product deconstruction and absorptive-capacity case studies that map high-level legal structures into field-level constraints.

First, the patent situation analysis focuses on climate-resilient molecular characteristics, especially those associated with abiotic stress resistance (drought, salinity, and heat). Using International Patent Classification (IPC) codes related to plant transformation and stress tolerance, such as, but not limited to, C12N15/82, our research scans patent applications in the past two decades.^61,62^ Instead of seeking absolute numerical exhaustiveness, the analysis focuses on patterns. The relative growth of filings regarding abiotic-stress-related traits, the growth of CRISPR-related plant patents, and the relative filing pattern of large multinational corporations and publicly funded research organizations are analyzed. Special attention is given to the ratio of the number of private and public patent holders in the most important areas like drought and salinity resistance, and the distribution of rights to a consortium of climate-prone states. The observations can be used to better understand the phenomenon of patent thickets, royalty layering, and market concentration, thus supporting the Market Power Effect that is explained in the following sections.

Second, a comparative legal study is performed to look at how TRIPS-plus commitments in regional trade agreements (RTAs) and bilateral free-trade agreements (FTAs) contribute to worsening or mitigating these structural imbalances. Line-by-line analysis of the IP chapters of a purposefully chosen sample of agreements, such as the United States–Mexico–Canada Agreement (USMCA), the Comprehensive and Progressive Agreement for Trans-Pacific Partnership (CPTPP), and selected North-South bilateral FTAs, is conducted to find provisions that: (i) require compliance with UPOV1991 or similar standards of plant variety protection (PVP); (ii) limit or reduce the privilege of farmers and exemption of breeders; and (iii) extend the term or scope of patents beyond the minimum standards in TRIPS. These clauses are coded based on their relative restrictiveness and put together to form an indicative regulatory stringency profile of each agreement. Though the study does not attempt to provide a formal econometric correlation, these profiles are addressed in the context of empirical indicators based on the existing literature and current WIPO and trade statistics, i.e. global plant variety application trends, the geographic distribution of plant-related IP, and seed export patterns, to demonstrate how high-quality protection can create the “Market Power Effect” and regulatory barriers to the diffusion of climate-ready technologies.

Third, we present a qualitative synthesis of institutional and legal obstacles to FTO experienced by public breeders and smaller organizations, especially in the Global South. Based on the analytical models of Dunwell,^21^ Kowalski et al.,^22^ Ferrari et al.,^41^ and Jarvie,^46^ we collect and organize the reported cases of patent thickets, cross-licensing conditions, and litigation practices in molecular plant technologies. This involves a survey of secondary sources, industry reports, and WIPO statistics on such aspects as trait licensing fees, compliance costs per variety, and the consolidation of corporate control in the proprietary seed market. These data examples are not seen as separate anecdotes, but as part of a larger thematic mapping of FTO constraints, thus depicting how transaction costs and legal uncertainty serve as *de facto* mechanisms of elimination of climate-resilient traits. A similar thread in this third layer deals with the regulation of DSI and the development of digital biopiracy. The study is a document-based analysis of policy submissions, negotiating texts, technical reports, and diplomatic statements issued under the Convention on Biological Diversity (CBD),^63^ the Nagoya Protocol,^7^ and the International Treaty on Plant Genetic Resources for Food and Agriculture (ITPGRFA).^64^ The primary instruments and the associated Secretariat documentation are publicly available, and the URLs are given in the reference list so that readers can consult the source texts directly. Instead of holding a formal Delphi panel, the analysis used a Delphi-style, iterative review of these materials to determine convergent and divergent positions of the parties on key questions, including: (a) whether DSI should be part of “Access and Benefit-Sharing” (ABS) regimes, (b) how to value digital-only features, and (c) how to administer multilateral benefit-sharing mechanisms. The study is based on institutional economics because it evaluates and discusses the administrative and transaction-cost results of alternative levy-based models, such as micro-taxes on seed sales, database access fees, and utilizes those analyses to inform the suggested DSI “Multilateral Fund.”

Fourth, to fill the gap between high-level IP structures and the realities on the ground of plant breeding, the study uses a product deconstruction approach based on Kowalski et al.^22^ Three typical climate-resistant varieties are chosen as case studies: a GM drought-tolerant maize, a gene-edited salt-tolerant rice, and a landrace-based hybrid. The component technologies of each archetype – such as promoters, selectable markers, transformation methods, gene-editing tools like the CRISPR-Cas9 complex, and specific trait-conferring sequences – are also identified, and their possible IP status and ownership patterns are inferred from the patent landscape insights described above. Special attention is given to the cumulative licensing burden, which occurs when several enabling technologies are managed by various rights owners. The most often given example of a situation where one salt-tolerant rice variety needs licenses with a dozen or more different patent holders is the result of this deconstruction exercise and is the direct inspiration of the humanitarian patent pools and segmented licensing models proposal.

Lastly, the study includes an institutional capacity and absorptive capacity evaluation for a group of climate-vulnerable developing nations. Based on the diffusion-of-innovation theory and the existing indicators of development, the study employs a limited number of proxies, including: (a) the approximate number of domestic molecular biologists per capita, (b) the presence and maturity of biosafety systems, (c) seed regulatory systems, (d) the presence of pro-bono or low-cost FTO legal services, (e) membership in international PVP and IP treaties, and (f) common capacity bottlenecks. These indicators are not provided as detailed country case studies, but rather as qualitative profiles, which show general trends––high reliance on imported molecular characteristics, low ability to clear products in-country, and large discrepancies between the formal commitments made by treaties and their actual implementation or enforcement. These profiles form the basis of the suggestion of decentralized “Regional Molecular Hubs” that integrate laboratory facilities with legal knowledge, especially to facilitate South-South cooperation in modifying climate-resilient characteristics for application in local agro-ecological settings.

## 4. Discussion: The Geopolitics of Molecular Adaptation – Navigating the IPR Thicket in an Era of Abiotic Stress

### 4.1. The Paradox of Precision: Technological Leap Vs. Biological Stagnation

The transition from selective breeding and classical hybridization to high-precision molecular breeding, introduced by CRISPR/Cas9 and other “New Genomic Techniques” (NGTs), has been announced as the final solution to the increasing challenges of abiotic stress in crops.^65^ It is worth keeping in mind the historical context. Farmers selected and re-sowed their best plants for roughly ten thousand years before any of this; the systematic crossing of chosen parent lines became a formal discipline only in the twentieth century; and the molecular era is a recent and short chapter in that longer history. Genome editing to confer drought, salinity, and heat tolerance has been done with unprecedented precision in laboratories.^66^ However, by the beginning of 2026, a major paradox had emerged. Although the list of possible climate-resistant traits is growing rapidly, their practical implementation in the fields is becoming slower.^67^ It is argued that the main obstacle is not a biological or technical one but rather in law, institutional framework, and economic issues. Rapela^29^ and Welsh et al.^38^ noted that academic and commercial capitalism in the late twentieth century, which together erased the line between state-funded research and commercial gain, has developed into a global patent thicket and a restraint on achieving further improvement of crop varieties. Modern molecular plants are not perceived as purely biological objects, but as complex collections of intellectual property.^68^ A single drought-tolerant soybean variety can be covered by more than thirty separate patents.^69,70^ The patents can contain very narrow aspects, including the gene promoter, the CRISPR guide RNA, the transformation protocol, and the eventual expression of the trait. It therefore follows that any breeder who wishes to move beyond an original idea to a commercial cultivar has to go through extensive freedom-to-operate audits, which often cost more than the scientific research itself.

### 4.2. Economic Reality: Market Power Versus Resilience

The empirical data provided by Galushko,^30^ together with seed-trade reporting available through the first quarter of 2026,^56,60^ show an alarming pattern: strong IPR regimes are more directly related to a “Market Power Effect” than to market expansion. Even though theoretical economic models suggest that IPR should trigger trade by protecting the assets of the exporter, the experience with abiotic stress-tolerant crop seeds is much more exclusionary. The most popular cultivars – those that can withstand heatwaves of 45°C or soil salinities of 15%—are limited to high-margin markets, mainly in North America, Brazil, and the European Union, where monopoly rents can be captured.^71^ This monopoly of blocking power^41^ makes the molecular instruments needed to achieve production of climate-resilient crops in areas, including the Indo-Malayan and Central Asian centers of crop diversity, economically inaccessible to the very countries that historically provided the ancestral genetic landraces (see Table 2).

**Table 2.** Economic barriers to abiotic stress mitigation (2026 estimates).

Economic Driver	Impact on Abiotic Stress Deployment
Monopoly Pricing	Smallholders in high-salinity regions are priced out of high-yield hybrids.^49^
Licensing Costs	High transaction costs deter mid-sized and public breeders.^41^
IP Consolidation	Four entities control 62% of proprietary stress-tolerant traits.^20^
Compliance Costs	The “Regulatory Wall” prevents orphan crop development.^47^

The figures in Table 2 are drawn from the sources cited and are reported as of the first quarter of 2026; the 2026 agricultural year was incomplete at the time of writing. The compliance figure is the average cost of discovery, development, and regulatory authorization for a single transgenic trait.^48^

### 4.3. Beyond North and South: China and the Middle-Income Innovators

We have so far used “Global North” and “Global South” as shorthand, and we should say plainly that this is a simplification that we adopt for convenience rather than one we would defend. The binary term maps technology ownership onto geography, and there are now several countries for which that binary mapping simply does not hold. China is the clearest case.

China is a center of origin for soybean, rice, and a number of millets, which places it on the resource-holding side of the binary distinction. It is also, on any measure, among the two largest investors in agricultural biotechnology in the world, with a state-directed program that has built substantial domestic capacity in genome editing and germplasm characterization. Under the national germplasm census launched in 2021, the Ministry of Agriculture and Rural Affairs surveyed some 625,000 villages and collected more than 530,000 new germplasm samples, a scale of public collection that no other country has attempted over the same period.^11^ China is therefore simultaneously a major supplier of genetic resources and a major generator of proprietary molecular technology.

Chinese policy has moved toward stronger IP protection rather than weaker. The fourth amendment to the Seed Law, in force from 1 March 2022, extended plant variety rights to harvested material, introduced the concept of essentially derived varieties, and raised the level of civil compensation available for infringement.^72^ China has not acceded to the 1991 Act of the UPOV Convention, but the amendment imports several of its central features, and the courts have been supplied with molecular identification standards, including DNA-fingerprinting protocols for a growing list of species, with which to apply them.^11,72^ Thus, the direction of IPR policy is toward the standards that this study describes as restrictive, adopted voluntarily, and for domestic reasons rather than imposed through a TRIPS-plus trade agreement.

The developments matter for our argument in three ways. First, it shows that the enclosure of plant genetic material is not something done simply to developing countries by developed ones. It is a policy choice that a large middle-income state is making on its own account, in pursuit of domestic seed-industry consolidation and food-security objectives of its own. Second, it complicates the benefit-sharing case, because a country that is at once a major provider and a major user of genetic resources has genuinely mixed incentives in a DSI negotiation, and the current stalemate at the CBD reflects that fact rather than any simple North–South standoff. Third, it suggests that reform should be framed by capability rather than by hemisphere. The distinction that actually matters is whether a given breeding program has the technical capacity to use a molecular tool and the legal capacity to clear the rights attached to it. On that axis, China sits with the technology holders; Brazil, Argentina, and India occupy an intermediate position, with strong public breeding sectors and growing patent portfolios; and much of Sub-Saharan Africa, along with the small island developing states, sits at the other end. Where we use “Global South” in what follows, it should be read as shorthand for the low-capability end of that spectrum and not as a claim about geography.

### 4.4. Molecular Advances: The CRISPR Surge and the Access Gap

In the last two years, the number of CRISPR-related plant patents has grown by 22% in the scientific community.^73,74^ At the same time, we have mapped quantitative trait loci for heat-shock proteins in wheat and for osmotic adjustment in rice, with a level of detail that would have been unimaginable ten years ago. However, as Jarvie^46^ observed, the legal tools of UPOV and TRIPS have failed to keep up with these breakthroughs. The innovation gap is increasing. The Global South has a limited amount of access to advanced molecular adaptations to abiotic stress, which is mostly because of the “Royalty Stacking phenomenon.^75^ ” Since a contemporary molecular plant uses many patented parts, the cumulative royalty payments, or stack, can account for a substantial portion of the total cost of the seed. To a farmer in a drought-ridden part of Sub-Saharan Africa, this makes the most drought-resistant seeds economically unfeasible, and he is forced to use older varieties that are less adapted and cannot withstand the current extremes of climate.

### 4.5. Sovereignty, Digital Sequence Information (DSI), and the New Biopiracy

One of the most important and urgent problems in 2026 is the emergence of DSI. The capability to sequence the genome of an extremophytic plant and patent its drought-tolerance genes without removing the plant from its natural habitat has brought in a new era of digital biopiracy, as Correa^76^ notes, now with modern metrics added. This tendency is a direct blow to the Nagoya Protocol and the ITPGRFA.^10^ Historically, sovereignty was associated with the physical plant. Digital synthesis is today circumventing the concept of sovereignty.^77^ Once the molecular blueprint of, for example, a salt-tolerant landrace from the Middle East is sequenced and potentially patented by a research institution in another jurisdiction, the existing benefit-sharing mechanisms intended to safeguard agrobiodiversity may face challenges in ensuring equitable recognition and distribution of benefits.

### 4.6. Institutional Failure: The Old Gothic Castle of Law

One of the most famous comparisons of the present IPR regime was made by Jarvie,^46^ who referred to the old Gothic castle as a magnificent structure that was not adapted to the needs of the present-day inhabitants. This analogy is especially suitable for abiotic stress crop breeding. We have constructed our existing laws (TRIPS, UPOV 91) around a world of physical seeds and traditional breeding methods and are struggling to govern a world of molecular parts and digital codes.^78^ The “Blocking Power,” which Ferrari and colleagues^41^ refer to, is not a commercial policy; it is a structural characteristic of the law. The legal system has enabled a few players to become gatekeepers to the global breeding of plants for climate response by permitting patents on general-purpose enabling technologies (including the CRISPR-Cas9 complex itself). Thus, if a university in India develops a heat-tolerant chickpea using a patented transformation procedure, it may be unable to commercialize this improvement without giving up a large share of the value to the holder of the patent on the technique, despite the strong genetic merits of the chickpea itself.

### 4.7. The “Black Swan” And Genetic Erosion

IP rights are high, which encourages “defensive breeding,” a breeding strategy that focuses innovation on a few high-value elite lines to guarantee patent security and predictability in the market.^29^ Although this practice maximizes short-term profit, it ultimately reduces the genetic base for the crop. We see the results in 2026: the world’s agriculture is more and more dependent on a small number of super-varieties, of which glyphosate-tolerant soybean and the stacked herbicide-tolerant and insect-resistant maize hybrids that dominate planted area in the Americas are the clearest examples.^79,80^ Even though they are designed to withstand particular stresses (e.g., drought), their relative genetic uniformity exposes them to Black Swan events, which are unanticipated combinations of stresses, e.g., a new pathogen arising during a record heatwave. Thus, the push toward uniformity mediated by IPR may prove a paradoxical way of making the global food system less resilient to unpredictable climate change.

### 4.8. The Way Forward: To a Humanitarian License and Utility Models

To close the growing gap between molecular capability and field-level reality, the academic community is moving toward “Utility Models” and “Humanitarian Licensing.”

1. Utility models: These models offer shorter-term (7–10 years) and less-complex protection of incremental innovations as proposed by Jarvie.^46^ This would be perfect for local breeders, who would adjust the molecular characteristics of globally distributed crop lines to the local soil and climatic conditions and would be able to guard their work without meeting the roughly USD 136 million cost of taking a fully patented trait through discovery, development, and regulatory authorization.^81^
2. Humanitarian Licensing: This is a model that was first introduced by organizations like the Public Intellectual Property Resource for Agriculture, a consortium established with support from the Rockefeller and McKnight Foundations to help public-sector institutions manage their agricultural IP for both commercial and humanitarian use,^34,82,83^ and the license can be segmented. That is, a patent holder has a monopoly in high-income markets and royalty-free licensing of noncommercial or subsistence use in low-income areas (see Table 3).

**Table 3.** Proposed shifts in the global ipr architecture (2026–2030).

Framework	Mechanism	Primary Goal	Feasibility (2026)
Open-Source Seed Initiative	No copyright for plant genetics; ensuring further use is not blocked.	Maintain the Genetic Commons	Moderate (Rising in Asia)
Utility Models	Reduced inventive step requirement for local adaptations.	Stimulate SME Innovation	High (Requires TRIPS reform)
DSI Benefit-Sharing Fund	A 1% levy on sales of seeds derived from digital sequences.	Compensate Centers of Diversity	Low (High Political Friction)
IP-Linked Sustainability	IPR protection is contingent on using Good Agricultural Practices.	Integrate IPM & Resilience	Emerging^20^

Table 3 sets out IPR proposals rather than findings. The feasibility ratings in the final column are our own qualitative judgment, based on the degree of treaty amendment that each mechanism would require and on the positions that parties have taken in the CBD and WTO negotiations discussed in sections 5.2 and 5.6.

### 4.9. Argument: The Need for Legal Resilience

We believe that without “Legal Resilience,” there is no way for “Molecular Resilience” to be attained. The current focus on maximizing appropriability, or the ability of a company to capture the value of an innovation, has been at the cost of dissemination. The value of a drought-resistant gene in a world where the climate crisis is manifested is not only the profit that it generates to a company, but also its potential to prevent famine and geopolitical turmoil in a region. Recent WIPO statistics^84^ show that open-source patent registrations have grown by 14%, whereas the transaction costs of cross-licensing of the key transgenic traits have grown by 12% per year. This implies that the Gothic castle is getting harder to navigate as we build more and more apartments in it. There is an urgent need to shift to a multilateral model, where the right to innovate to be climate resilient is prioritized over the veto of existing patent holders.

### 4.10. Beyond the Patent Thicket

The molecularly bred crop plant is an important way that humanity adapts to a warming planet. However, if the legal system remains a zero-sum game between the holders of technology and the holders of genetic resources, then the biological effectiveness of crop research will be lost. The high price of IP enforcement and the market-power effect both contribute to a situation where we have molecular solutions to abiotic stress, but are not legally determined to use them. An IP regime redesign by a group of scholars is no longer just a scholarly quest for fairness–it becomes a basic requirement for international biosecurity. Ray and colleagues^85^ calculate that global yields of maize, rice, wheat, and soybean are currently rising at 1.6%, 1.0%, 0.9%, and 1.3% per year, respectively, well short of the roughly 2.4% per year that would be required to double global crop production by 2050. To close that gap, we must move from a paradigm of appropriability to one of adaptation. Without such a change, the most refined molecular crops will be restricted to the Gothic halls of our own legal architecture, and the areas to which they were designed to apply will fall into ruins.

## 5. Recommendation

### 5.1. Strategic Reform of the IPR Architecture and the Utility Model Tier

The current global IP system for molecular plant science mainly offers two options: very strong utility patents and relatively weak plant-variety protections.^86^ This limited either–or structure makes it difficult to introduce drought- and salinity-tolerant traits in countries of the Global South. To fill this gap, a tiered legal resilience framework would need to be taken up at two levels at once. The first would be multilateral: WIPO, the WTO TRIPS Council, and UPOV set the standards within which national systems operate. The second would be national: legislatures and patent offices are the bodies that actually grant, refuse, and enforce rights, and nothing changes within a country until they act. Neither level works alone. A multilateral instrument without domestic implementing legislation is a statement of intent, and a single country adopting a utility-model tier on its own would find its breeders still blocked by foreign patents the moment that they try to export. Such a tiered framework would put more emphasis on the quick spread of climate-adaptative technologies than on the long-term extraction of monopoly rents. One of the key suggestions is the official institutionalization of a Climate-Utility Model at the level of WIPO, which is specifically aimed at incremental innovations in abiotic-stress tolerance. This model would offer a shorter protection period and a lower threshold to innovation, unlike full utility patents, which require a high threshold for the inventive step and grant twenty years of exclusivity. This, in turn, would allow public research institutions in the Global South to protect their own local adaptations – such as a gene-edited version of African yam adapted to unique alkaline soils – without being bound by the gothic castle of primary transformation patents owned by multinational corporations. We encourage a flood of localized secondary innovation that is currently suppressed by the prohibitive price of legal compliance and patent registration by lowering the transaction costs of entry into the formal intellectual-property system.

### 5.2. The Multilateral Benefit-Sharing Solution to the DSI Stalemate

With the process of dematerialization that molecular biology is experiencing, whereby physical seeds are replaced by DSI as the research medium of choice, the bilateral benefit-sharing systems of the Nagoya Protocol have become obsolete in principle. Current research does not offer a practical way to both keep research open to everyone and respect megadiverse countries’ control over their own genetic resources. To address this gap, a global DSI Multilateral Fund must be created, funded by a small tax on the commercial sale of any seed variety the developmental lineage of which includes DNA sequences patented under the international multilateral IPR system. This method reduces the administrative cost of tracing individual, virtually untraceable digital sequences through the use of a practical output-based model of taxation. How the income is disbursed matters at least as much as how it is raised, and we want to be specific about this because the point is not a small one. If receipts were paid into national treasuries, they would be absorbed into general revenue and lost to the purpose for which they were collected. We therefore propose that the fund disburse directly to relevant recipients rather than to governments: national and regional genebanks, public breeding institutes, and registered community seed organizations in the countries of origin, through an audited grant window administered by the fund itself. Ministries of agriculture would sit on the governing board and approve country programs, but would neither receive nor hold the money. This would be an effective way of internalizing the ecological debt of the world agriculture-biotechnology industry to the traditional communities whose ancestral seeds are the raw genetic scripts of the modern molecular innovations, thus establishing a sustainable cycle whereby commercial success directly funds the conservation of the raw materials for future innovation.

### 5.3. Reducing Patent Thickets with Compulsory Humanitarian Pools

The emergence of patent thickets in the field of New Genomic Techniques (NGTs), including CRISPR-Cas9, has led to the situation where one type of technology might have dozens of licenses, which creates a royalty stacking effect, making climate-resistant seeds unaffordable to smallholder farmers.^87^ To break these obstacles, the global community should require the establishment of Abiotic-Stress Patent Pools that will be used for humanitarian purposes. These pools would work along the lines of the Medicines Patent Pool (MPP)^79^, a United Nations-backed organization founded by Unitaid in 2010 which negotiates voluntary licenses from patent holders on treatments for HIV, hepatitis C, tuberculosis and, more recently, COVID-19, and then sub-licenses them to generic manufacturers for supply in low- and middle-income countries. On the same principle, an agricultural patent pool would bring together the necessary enabling technologies, promoters, selectable markers, structural genes, and gene-editing enzymes into one simplified licensing organization. Involvement in such pools must be associated with corporate Environmental, Social, and Governance (ESG) reporting and governmental research and development subsidies, which would give large IP holders a reason to grant developing-country breeders access to their core patents in exchange for tax credits or preferential regulatory treatment of their high-margin products. We propose access rather than outright donation, and the distinction is deliberate. Rights holders will resist surrendering title to foundational patents and are unlikely ever to agree to it, but the record of the Medicines Patent Pool suggests that they will license into a pool on defined terms when the markets concerned are ones that they were not going to serve at commercial rates in any case.^88^ The resulting licenses must be royalty-free on subsistence crops and orphan species in least developed countries, but be of normal commercial terms on commodity crops in industrialized markets. This type of market segmentation is used to ensure that the market power seen in trade studies does not threaten the survival of people living in areas with severe food insecurity.

### 5.4. Redesigning Institutional Capacity and Diffusion of Absorptive Capacity

The suggested reform is just a mere theoretical exercise if the target countries do not have the absorptive capacity to utilize molecular tools. The existing development models are ineffective, as they focus on the transfer of technology and fail to consider the regulatory wall that does not allow locally targeted products to reach the field. Thus, it is necessary to invest heavily in decentralized “Regional Molecular Hubs” that integrate laboratory facilities with pro-bono FTO legal services. These centers would be centers of South-South cooperation, where scientists in different parts of the world with similar agro-ecological problems, like high salinity in the Nile Delta and the Mekong Delta, can share biosafety information and work through the patent status of proprietary technologies within a common legal framework. These hubs would lower the entry barrier of local small and medium enterprises and state universities by centralizing the legal and technical deconstruction of complex transgenic and gene-edited products. This strategy shifts the global innovation model from a top-down model of diffusion of proprietary seeds to a polycentric model where innovation is co-produced and locally tested, so that the resulting products are biologically and legally compatible with the ecosystems, agricultural systems, and climates that they are intended to support.

### 5.5. Genetic Uniformity and the Case for Supporting Existing Genebanks

The modern tendency toward genetic homogeneity of crops, which is fueled by the commercial need to have stable and high-performing proprietary varieties, is a systemic threat to food security in the world that has not been considered adequately in the context of IP debates. In an earlier version of this argument, we proposed a “Genetic Breadth Requirement,” under which patent offices would assess a diversity impact statement attached to each new application. On further reflection, we do not believe this is workable, and we set out why. There is no agreed metric for genetic breadth. The question of whether it should be measured within a line, across a breeder’s portfolio, across a breeding station, or across a national collection has no settled answer, and the four measures would give different results for the same application. For crops that are effectively inbred, which includes wheat, rice, and soybean, low within-line diversity is a deliberate and necessary property rather than a defect, so a breadth test would penalize exactly the varieties whose uniformity is agronomically justified. It is also unclear which authority would carry out the assessment. Patent examiners are trained in claim construction and prior art, not in population genetics and selective breeding, and no examining office currently has the capacity to make judgments of this kind at scale.

We therefore propose a different route to the same objective, and one built on institutions that already exist rather than on a new regulatory test. Conservation is already carried out by roughly 1,750 genebanks holding on the order of 7.4 million accessions worldwide. Among them are the eleven CGIAR collections and the Svalbard Global Seed Vault, which holds duplicate samples deposited by more than fifty genebanks.^89,90^ The problem with this network is not that it is missing, but rather that it is chronically underfunded, and much of the network sits in precisely the centers of diversity that this paper is concerned with. A “Tax-for-Traits” levy, under which firms commercializing traits derived from indigenous landraces contribute a defined share of the resulting revenue, should therefore be directed to the long-term endowment of these existing genebanks and of the community seed systems that supply them. This is administratively simpler than a genetic breadth requirement, is enforceable through conservation agreements that are already in force, and does not ask patent offices to do something they are not staffed to do. It also keeps the underlying principle intact: agrobiodiversity is not a free public good, but a maintained global heritage that costs money to maintain, and that money has to come from somewhere.

### 5.6. The Case of a Climate Emergency Waiver and Antitrust Oversight

The scale of projected climate-induced yield losses calls for a fundamental shift in how international trade law is understood, moving toward a World Trade Organization–level Climate Emergency Declaration that recognizes essential agricultural characteristics. This statement would allow the mandatory licensing of certain molecular technologies in case of national food crises, comparable to the Ministerial Decision on the TRIPS Agreement adopted at the WTO’s Twelfth Ministerial Conference in June 2022,^91^ which gave eligible members a time-limited waiver of Article 31(f) so that they could authorize the use of patented subject matter for the production and export of COVID-19 vaccines. That precedent is instructive in two directions. It shows that the membership will act on public-health grounds when the case is pressed hard enough. It also shows the limits of what can be agreed, since the negotiated text was considerably narrower than the original proposal and the promised extension to therapeutics and diagnostics was never concluded. Also, there is a need for increased antitrust regulation in the international seed market to avoid IP consolidation that creates a market-power effect that limits exports to maintain monopoly pricing. National and regional competition-regulating authorities must pay attention to the blocking capability of crop patents. The bodies that we have in mind are the antitrust and merger-control agencies with jurisdiction over the seed sector, principally the Antitrust Division of the United States Department of Justice and the Federal Trade Commission, the European Commission’s Directorate-General for Competition, China’s State Administration for Market Regulation, and Brazil’s CADE, together with the International Competition Network^92^ through which these agencies share analysis. We should be clear that the WTO is not among them. It is not a competition-regulating authority; it cannot review a merger or an exclusionary licensing practice, and its role in our proposal is confined to the trade-law question discussed immediately above. That the necessary institutional capacity already exists is evident from the review of the Bayer–Monsanto and Dow–DuPont transactions, in which several of these agencies examined the effect of consolidation on innovation and required substantial divestitures as a condition of clearance.^93^ Treating the genetic scripts of drought and heat tolerance as an essential facility, international law can require access to these tools, thus ensuring that the molecular basis of our food system is a shared heritage of all humanity instead of a property of a few, thus closing the divide between the incentive of the market and the human right to food in a warming world.

## 6. Conclusion

This paper argues that, since climate change is making weather more unpredictable, the future of world food systems cannot be based on the current dichotomy of private property and shared commons. Essentially, genetic resilience would cease to be a commercial product, but rather be a facility that is vital to human security. The current legal structure, characterized by hard utility patents and trade-ratchet systems, has created a structural bottleneck that impedes the transfer of molecular innovations of crops to the areas where they are most in need. To move out of the state of legal stalemate to an institutional stability, it is possible to adopt a polycentric system, which would balance the commercial interests of patent holders against humanitarian needs. The results of this paper are united by two different but interrelated crises: (a) the statistical concentration of ownership of the molecular genetic variations underlying key environmental tolerance traits, and (b) the legal dematerialization of biology. We have carefully mapped patent thickets to prove that the market-power effect is not just a theoretical concept but a measurable reality.

When a single climate-ready variety needs a dozen licenses just to be planted, the system rewards exclusion instead of innovation. Also, our interpretation of TRIPS-plus provisions shows that there is a systematic constriction of the legal flexibilities that were initially meant to safeguard developing countries. All these forces together form a Gothic Castle of IP law, a building that can protect corporate investment, but in the end, does not serve the interests of the global population in times of environmental crisis.

Our findings show an urgent need for a climate emergency waiver and regional molecular hubs. We have demonstrated that the regulatory wall is, in many cases, more daunting than the technical difficulties of gene editing. The Global South will continue to be in a state of technological dependency without a legal break-glass mechanism at the WTO level and localized centers of technical-legal expertise. The way out of this is to propose tiered patent protections, including utility models and humanitarian patent pools, in our proposed polycentric model. These instruments are not aimed at eliminating IP, but rather at rebalancing it, so that the molecular basis of our food system, the drought- or heat-tolerance genes and salt-resistant promoters, is not treated as the exclusive property of a small number of multinational corporations and patent holders. Finally, IP law should be changed to accommodate the dynamic and digital character of modern biology. Continuing to apply twentieth-century mechanical thinking in the molecular age is likely to contribute to a reduction in yields that no court case can remedy. The shift to a molecular commons is not a form of charity, but a rational requirement of a humanity that has a common, interconnected food system. We need to decide whether to retain the “Gothic Castle” of our modern IP regime that excludes the most vulnerable farmers in the world. The future of food security lies in the capacity to change the legal structure of IP ownership into a cooperative survival mechanism, and in doing so to share the genetic basis of crop resilience, both the genetic variants themselves and the tools needed to deploy them, in the best interest of humanity.

## Data Availability

The original contributions presented in this study are included in the article. Further inquiries can be directed to the corresponding author.

## Abbreviations

ABS: Access and Benefit-Sharing
CAGR: Compound Annual Growth Rate
CBD: Convention on Biological Diversity
CPTPP: Comprehensive and Progressive Agreement for Trans-Pacific Partnership
CRISPR / CRISPR-Cas9: Clustered Regularly Interspaced Short Palindromic Repeats / CRISPR-associated protein 9
CVC: The Regents of the University of California, the University of Vienna, and Emmanuelle Charpentier (joint CRISPR patent applicants)
DSI: Digital Sequence Information
ESG: Environmental, Social, and Governance
FAO: Food and Agriculture Organization of the United Nations
FTA / FTAs: Free-Trade Agreement(s)
FTO: Freedom to Operate
GM: Genetically Modified (crops/seeds)
GMO: Genetically Modified Organism
IP: Intellectual Property
IPC: International Patent Classification
IPR / IPRs: Intellectual Property Right(s)
ITPGRFA: International Treaty on Plant Genetic Resources for Food and Agriculture
MPP: Medicines Patent Pool
NGT / NGTs: New Genomic Technique(s)
PIPRA: Public Intellectual Property Resource for Agriculture
PTAB: Patent Trial and Appeal Board (United States)
PVP: Plant Variety Protection
RTA / RTAs: Regional Trade Agreement(s)
TRIPS: Agreement on Trade-Related Aspects of Intellectual Property Rights
TRIPS-plus: Provisions in trade agreements that exceed the minimum standards set by TRIPS
UPOV / UPOV 1991: International Union for the Protection of New Varieties of Plants (1991 Act)
USMCA: United States–Mexico–Canada Agreement
WIPO: World Intellectual Property Organization
WIPO IPSTAT: WIPO Intellectual Property Statistics database
WTO: World Trade Organization
